# Turkish perlite supported nickel oxide as the heterogeneous acid catalyst for a series of Claisen–Schmidt condensation reactions

**DOI:** 10.3906/kim-2010-42

**Published:** 2021-08-27

**Authors:** Sakshi Kabra MALPANI, Deepti GOYAL, Stuti KATARA, Ashu RANI

**Affiliations:** 1 Department of Chemistry and Biochemistry, Faculty of Physical Science, Jyoti Nivas College Autonomous, Bengaluru, Karnataka India; 2 Department of Applied Chemistry, School of Vocational Studies & Applied Sciences, Gautam Buddha University, Greater Noida, UP India; 3 Department of Pure and Applied Chemistry, Faculty of Science, University of Kota, Kota, Rajasthan India

**Keywords:** Turkish perlite, nickel oxide, solid acid catalyst, Claisen–Schmidt condensation, 2,6-bis(substituted benzylidene)cyclohexanones

## Abstract

Potentially active and eco-friendly solid acid catalysts have been synthesized by loading different weight percentages (10, 15, and 50) of nickel oxide on thermally activated Turkish perlite through the deposition-precipitation method. Structural features of prepared catalysts were analyzed using BET surface area analysis, X-ray diffraction, scanning electron microscope (SEM), SEM-EDX, transmission electron microscopy (TEM), Fourier-transform infrared (FT-IR), pyridine adsorbed FT-IR, UV-Vis diffuse reflectance spectroscopy (DRS), and thermogravimetric analysis (TGA) techniques. Pyridine adsorbed FT-IR analysis confirmed the presence of the optimum amount of Bronsted acidic sites in a catalyst having 15 wt. % loading of nickel oxide, which was tested for catalyzing a series of Claisen–Schmidt condensation of cyclohexanone and aromatic aldehydes to produce good isolated yield (90%–93%) of 2,6-bis(substituted benzylidene)cyclohexanones, significantly used in anti-tumor and cytotoxic activities. The high catalytic efficiency of the chosen catalyst remains almost intact up to six reaction cycles. On higher wt. % loading of nickel oxide, crystallite size increases along with agglomeration of larger nickel oxide particles on catalyst surface resulting in pore blockage and poor catalytic activity. Loading of NiO on the surface of thermally activated Turkish perlite was confirmed by SEM-EDX analysis, and TEM observations show that the particle size of the preferred catalyst was less than 50 nm. Based on results drawn from XRD, FT-IR, pyridine adsorbed FT-IR, UV-Vis DRS studies, model structures were proposed for Turkish perlite and all prepared catalysts. During this work, the catalytic potential of the preferred catalyst was compared with other previously reported catalysts, and it showed appreciable results. The formed products were further confirmed by their melting point and ^1^H-NMR analysis.

## 1. Introduction

Acid catalysis is an important area of organic synthesis and vital industrial significance predominantly in the petrochemical, fine chemical, and pharmaceutical industries [1]. Many intrinsic drawbacks of the use of homogeneous acid catalysts have stimulated the research for recyclable strong solid acids to replace conventional, toxic, homogeneous acid catalysts. Heterogeneous catalysts such as phosphosulfonic acid, nano sulfated zirconia, and boric acid[2–4]are being utilized as a replacement of homogeneous acids in industrially important reactions. As a lucrative alternative to classical solid catalysts, the use of economical solid catalysts like activated fly ash precipitated silica catalyst, sulfated zirconia/fly ash, cerium triflate/fly ash, scandium triflate/fly ash, phosphomolybdic acid/fly ash, fly ash solid base catalyst[5–10], and other waste-derived catalysts[11–15]is gaining interest nowadays.

Perlite is a hydrated, pozzolanic, naturally occurring volcanic glass formed by the cooling of volcanic eruptions, estimating about 1.1 million metric tons production in Turkey [16]. It has a layered structure whose skeleton mainly comprises oxides of silicon, aluminium, potassium, and sodium, while oxides of titanium, calcium, magnesium, iron, and chemically combined water, etc. are present in small quantities [17]. Its particles are neutral, fluffy, non-toxic, can expand up to 20 times than their original volume on heating beyond 900 °C. Its particle size and specific surface area are 0.2‒4 mm and 1.22 m^2^g^–1^ respectively [18,19].It is mainly used in producing construction materials, adsorption and removal of atmospheric pollutants, horticulture, etc. [20–22], while its application in heterogeneous catalysis is not much explored in past except only a few reports [23–30]. Turkey is the third worldwide leading producer of perlite and accounts for about 16% of its total production [14].It is, thus, assumed that Turkish perlite, which has high silica and alumina composition might possess significant stable surface-active sites and can be converted into a potential heterogeneous acid catalyst for catalyzing industrially beneficial organic transformations. Nickel-based catalysts supported on different substrates like silica, alumina, zeolites have attracted research attention because of their potential applications in many important reactions such as hydrogenation, diesel steam reforming, ozonation of 2,4-dichlorophenoxyacetic acid, dry reforming of methane[31–34], etc. Particularly, supported nickel oxide catalysts have been utilized in numerous potential reactions such as hydrogen production, oxidative dehydrogenation, hydrogen peroxide decomposition, etc. [35–37], while fewer records are found for its application in condensation reactions [38]. Catalyst support is vital as it facilitates higher dispersion of nickel oxide and prevents its aggregation[39], which, in turn, enhances the efficiency of catalyst, and, thus, maximum conversion and yield % of desired products are achieved. With the increasing social interests over environmental degradation and future resources, it is of great importance for chemists to come up with new approaches that are less hazardous to human health and the environment. In this series, we have tried to explore stable surface-active sites of a novel silica-alumina enriched natural waste- Turkish perlite and its efficiency as catalyst support. 

In the current work, a novel, efficient Turkish perlite supported nickel oxide catalyst (NPC-15) was prepared by loading nickel oxide on thermally activated Turkish perlite (TAP), and it successfully catalyzed a series of Claisen–Schmidt condensation reactions giving a higher isolated yield % of desired products, 2,6-bis(substituted benzylidene)cyclohexanones, used in anti-tumor, anti-cancer, and cytotoxic activities[40]and also serve as an important precursor for the synthesis of potentially bioactive pyrimidine derivatives [41, 42].Table 1 summarizes some nickel catalysts supported on economical substrates already precedented in literature, utilized in different reactions including Turkish perlite supported nickel oxide catalyst (NPC-15) presented in this work. Our work is different and innovative in terms of sustainability and reusability of NPC-15 up to six consecutive reaction cycles, also the catalytic potential of NPC-15 was tested for a series of Claisen–Schmidt condensation reactions, which is quite an uncommon field of catalysis for supported nickel oxide catalysts. Use of less expensive support, recyclable catalyst, solvent-less reaction conditions, green methodology, ease of product purification are the key features of this protocol, and, thus, it may be considered as an efficient alternative to the existing, high-priced, less-effective procedures. This work is aimed to act as a stepping-stone for the prospective researchers into the rewarding field of utilization of waste materials like Turkish perlite as the catalyst support in the development of heterogeneous catalysts.

**Table 1:** Various nickel catalysts supported on different, economical substrates.

Name of catalysts	Supports used	Chemicals used	Method used for catalyst synthesis	Catalytic applications
NiFAk [37]	Fly ash	Ni(NO3)2.6H2O, CaO,urea	Microwave assisted solution combustion	H2O2 decomposition
NiPCH [36]	Porous clay heterostructures	Ni(NO3)2.6H2O, oxalic acid	Evaporation	Oxidative dehydrogenation of ethane
Ni/SiO2-RHA [43]	Rice husk ash	Ni(NO3)2.6H2O, aq. NH3	Ion exchange	-
Ni-10/AC [44]	Activated carbon	Ni(NO3)2.6H2O	Wet impregnation	Benzaldehyde hydrogenation
Ni/PF-B [26]	Perlite treated with Na2CO3	Ni(NO3)2.6H2O and Mg(NO3)2.6H2O, solid anhydrous Na2CO3	Co-precipitation followed by reduction	Sunflower oilhydrogenation
Nickel boride catalyst [45]	Hydrothe-rmal modified perlite	NiCH3(CO2)2.4H2O, NaBH4, NaOH	Impregnation	Hydrogenation of nitrobenzene
NPC-15 [This study]	Turkish perlite	Ni(NO3)2.6H2O, 6 M NaOH solution	Deposition-precipitation	Series of Claisen-Schmidt condensation reactions

## 2. Expreimental

### 2.1. Chemicals

Nickel nitrate, sodium hydroxide, nickel oxide, cyclohexanone, and aromatic aldehydes were purchased from Sigma Aldrich, Bengaluru, Karnataka, India. Turkish perlite was supplied by Indica Chem. Ind. Pvt. Ltd., Kotdwar, Uttarakhand, India.

### 2.2. Catalyst synthesis

**Scheme 1:**
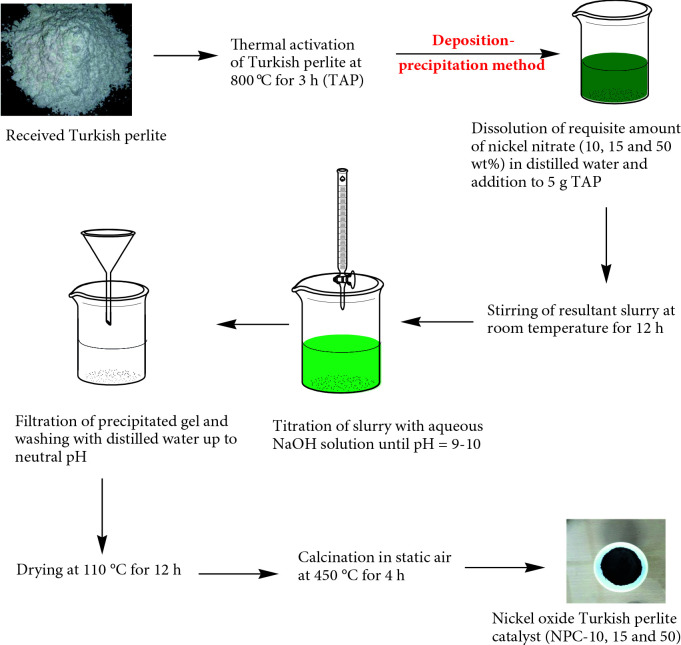
Synthesis of nickel oxide Turkish perlite catalyst.

After being received, Turkish perlite was initially activated thermally at 800 °C for 3 h to remove moisture, carbon, and other impurities to form TAP (thermally activated perlite). All the catalysts investigated in this study (NPC-10, 15, and 50) were synthesized by loading 10, 15, and 50 wt.% nickel oxide on TAP by deposition-precipitation method, using NaOH as a basification agent. The required amounts (1.97 g for 10 wt. %, 2.95 g for 15 wt. %, 9.83 g for 50 wt. %) of precursor salt (nickel nitrate) in an aqueous solution phase were mixed with 5 g of TAP and agitated for 12 h at room temperature. Thus, obtained homogeneous mixture of metal salt and TAP was treated with 6 M NaOH solution until pH value becomes 9–10. Then, formed precipitated gel was filtered, washed several times up to neutral pH, dried at 110 °C for 12 h, and then calcined in static air at 450 °C for 4 h. The negative results of Dimethylglyoxime (DMG) tests confirm that no nickel ions leached off from the catalysts during their synthesis. The synthesis process of NPC catalyst is presented in Scheme 1. 

### 2.3. Catalyst characterization

Structural and morphological features of all catalytic materials were analyzed by BET surface area analysis (Anton Paar India Pvt. Ltd., Gurgaon, Haryana, India), XRD (Bruker, Mumbai, Maharashtra, India), SEM, SEM-EDX JEOL India Pvt. Ltd. Mumbai, Maharashtra, India.), TEM (Hitachi High-Tech India Private Limited, Mumbai, Maharashtra, India), FT-IR, pyridine adsorbed FT-IR (Bruker, Mumbai, Maharashtra, India), UV-Vis DRS (Perkin Elmer (India) Pvt Ltd., New Delhi, India) and TGA (Mettler Toledo) techniques.^1^H NMR spectra of products were recorded on Bruker DRX300 spectrometer.

### 2.4. Catalyst evaluation

**Scheme 2:**
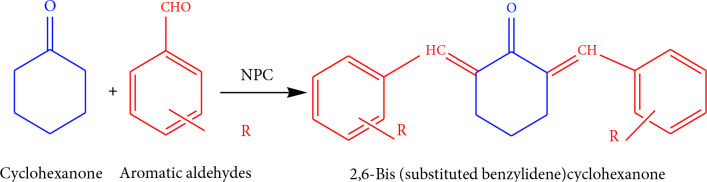
Condensation of cyclohexanone and aromatic aldehydes over NPC to give 2,6-bis(substituted benzylidene) cyclohexanone.

The condensation reactions (Scheme 2) catalyzed by NPC-10, 15, and 50 were performed in a liquid phase batch reactor equipped with 250 mL RBF (round bottom flask), magnetic stirrer, and spiral glass condenser, placed in a thermostat. In this procedure, a mixture of cyclohexanone and aromatic aldehydes (molar ratio = 1:2) was placed in a RBF and mixed with the activated catalyst. The reaction mixture was stirred at different times and temperatures. Once the reaction is over, the catalyst was removed from the reaction mixture by simple filtration. Melting points of products were detected by using Melting Point System MP30, Mettler Toledo India Pvt. Ltd., Vasai, Maharashtra, India, melting apparatus.

## 3. Results and discussion

### 3.1. Characterization of catalysts

Table 2 shows the surface properties of Turkish perlite and NPC-10, 15, and 50 catalysts with different nickel oxide loadings (10, 15, and 50 wt. %). The results indicate that when the nickel oxide loading increased from 10 to 15 wt. % in NPC catalyst, a decrease in the surface area was observed. Further increase in nickel oxide loading resulted in a significant decrease in surface area, which may be due to the blockage of pores of Turkish perlite by nickel oxide dispersion. Thus, from these results, it can be established that an increase in nickel oxide loading is responsible for the agglomeration of nickel oxide species to form bigger particles, and, thus, their diffusion decreases. These results are in good agreement with XRD results. Despite this, it was also observed that in NPC‒10 and 15 the crystallite size remains unaltered, but, on further increase in nickel oxide loading, crystallite size also increased. The above discussion indicated that higher wt. % loading of nickel oxide does not provide sufficient active sites due to their agglomeration as inferred from increased crystallite size [46,47].

**Table 2:** BET specific surface area of samples.

Samples	BET specific surface area(m2/g)
Turkish perlite	2.3
NPC-10	2.2
NPC-15	1.9
NPC-50	1.1

**Figure 1:**
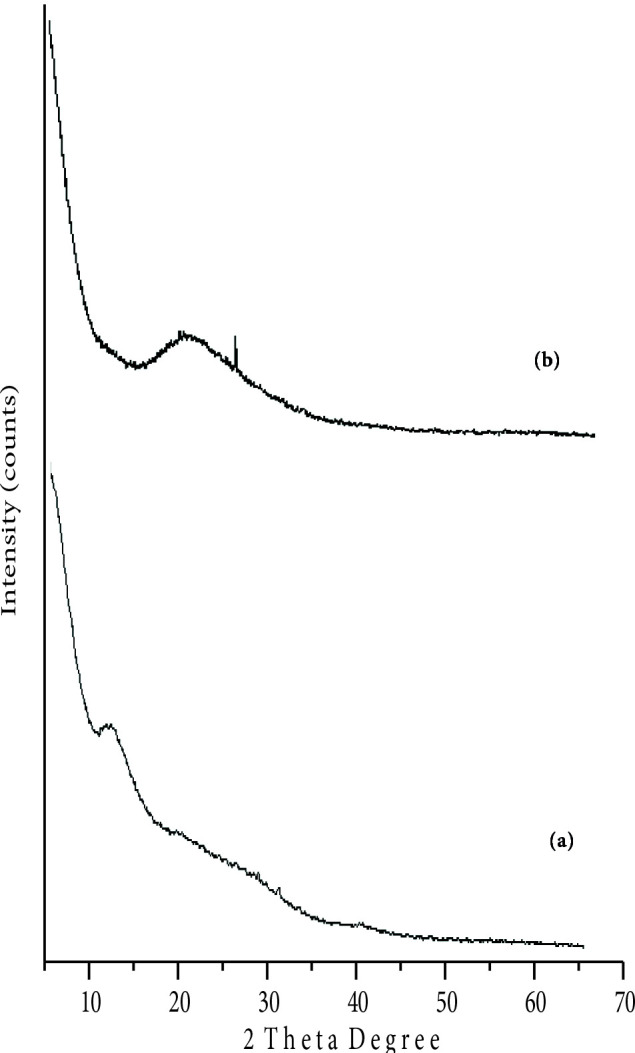
X-ray diffraction patterns of (a) Turkish perlite and (b) TAP.

**Figure 2:**
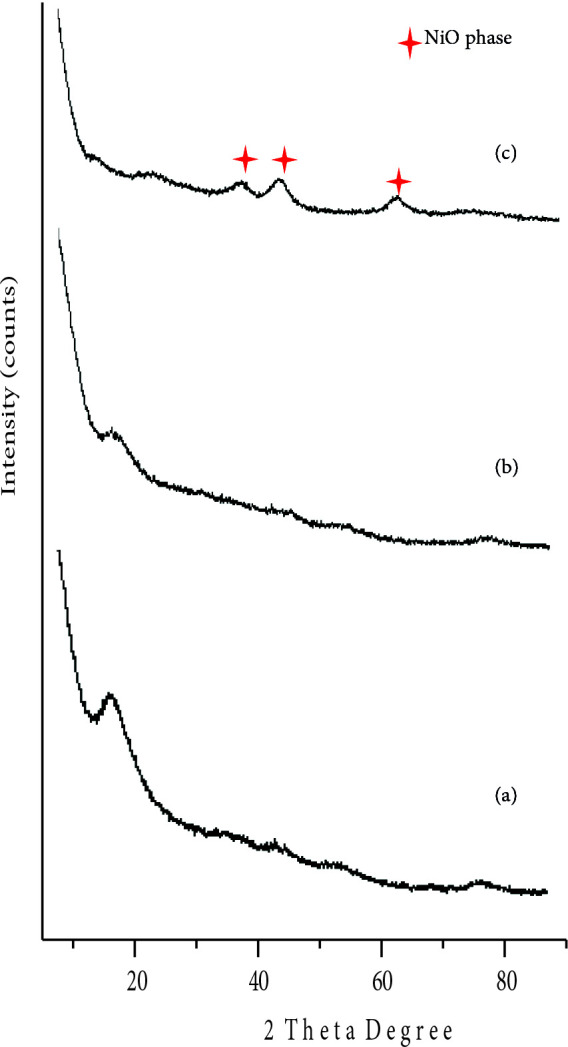
X-ray diffraction patterns of (a) NPC‒10, (b) NPC‒15 and (c) NPC‒50.

The XRD of Turkish perlite (Figure 1a) shows the existence of a hump between 2θ = 10‒15° attenuated to amorphous silica [48].However, in TAP, a small crystalline peak appears at 2θ = 27.642° (Figure 1b) which confirms the formation of quartz crystalline phase in the sample on heating[49]along with a broad hump at 2θ = 22‒23° showing amorphous content of silica [50,51]. The absence of crystalline peaks in X-ray diffraction patterns of NPC‒10 and 15 (Figures 2a and 2b) is due to the dispersion of nickel oxide particles on the Turkish perlite surface in the amorphous phase and tiny in size to give an XRD peak[52] in lower wt. % catalysts. While, in case of NPC‒50 (Figure 2c), small peaks begin to appear at 2θ = 37.26, 43.29, 62.88° corresponding to Miller indices [003], [012] and a combination of the [104] and [110] reflections of nickel oxide, respectively[53], which are evident of formation of Ni‒O phase in the sample. However, because of the weak, broad, and partially overlapping peaks, the crystallite size determination of the nickel oxide containing phase is difficult in NPC‒50 [54].

**Figure 3:**
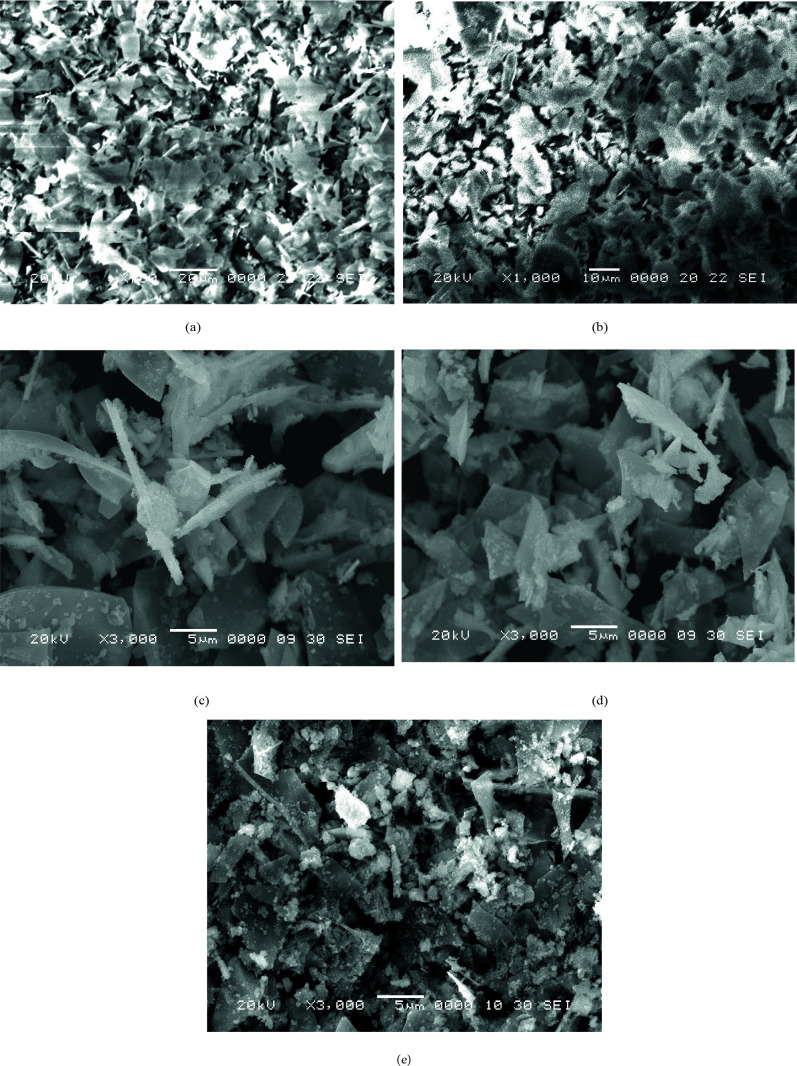
SEM micrographs of (a)Turkish perlite, (b) TAP, (c) NPC‒10, (d) NPC‒15 and (e) NPC‒50.

SEM micrograph of Turkish perlite (Figure 3a) revealed randomly placed irregular shred particles with uneven edges, open pores also shown in other reports [55, 56].SEM image of TAP (Figure 3b) is mainly fragmatic, glassy, and random[57],but the morphology is less irregular indicating removal of water at a high temperature, which leads to a reduction in porosity and also agglomeration of smaller particles. SEM images of NPC‒10, 15, and 50 (Figures 3c, 3d, 3e) demonstrate the dispersion of shiny, fine nickel oxide particles on the surface of Turkish perlite. It was also found that the porosity of Turkish perlite particles was decreased on the loading of nickel oxide.

EDX analysis of Turkish perlite, NPC‒10, 15, and 50 (Table 3) shows the presence of SiO_2_, Al_2_O_3_, K_2_O, Na_2_O, and other minor metal oxides in the samples. However, the presence of nickel oxide in all NPC catalysts confirms its effective loading on the Turkish perlite surface. 

**Table 3:** EDX analysis of Turkish perlite, NPC‒10, NPC‒15, and NPC‒50.

Samples	SiO2 (wt%)	Al2O3 (wt%)	K2O (wt%)	Na2O (wt%)	ZnO (wt%)	FeO (wt%)	NiO (wt%)	LOI
Turkish perlite	72.74	14.79	7.48	2.10	2.04	0.91	-	4.1
NPC‒10	71.09	12.14	5.68	1.86	-	-	8.42	1.2
NPC‒15	67.40	11.69	5.24	1.78	-	-	13.89	0.8
NPC‒50	40.28	9.47	-	1.54	-	-	48.69	0.24

**Figure 4:**
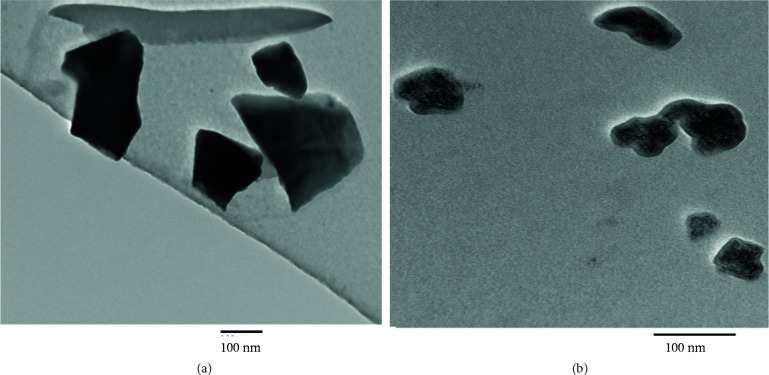
TEM micrographs of (a) Turkish perlite and (b) NPC‒15.

A more realistic vision of irregular morphology, ragged shreds of Turkish perlite can be furnished by its TEM image (Figure 4a). The micrograph of NPC‒15 (Figure 4b) shows fine dispersion of nickel oxide particles on the Turkish perlite surface. These results are in agreement with SEM images. TEM observations also show that the particle size of NPC‒15 is less than 50 nm.

**Figure 5:**
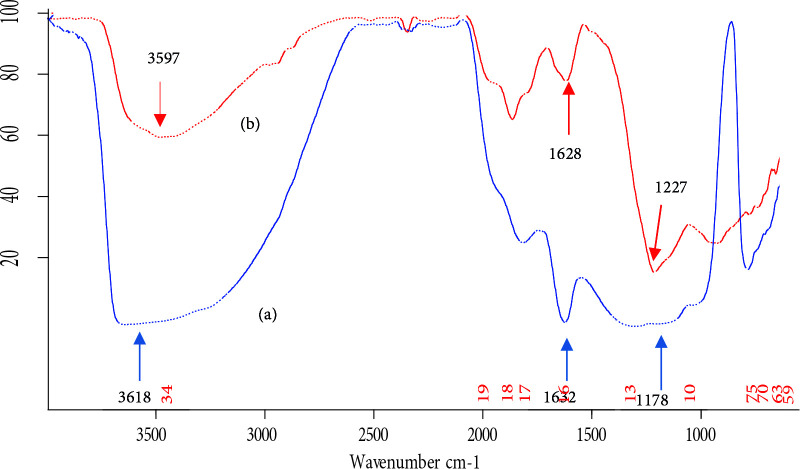
FT‒IR spectra of (a) Turkish perlite and (b) TAP.

FT-IR spectrum of Turkish perlite is shown in Figure 5a. A broad band in the range between 3700‒3000 cm^‒1 ^shows the presence of surfacial hydroxyl groups (–Si-OH) with strong intermolecular hydrogen bonding and physisorbed water molecules [58,59].The intensity and broadness of this band diminished in TAP (Figure 5b), which is due to the loss of water as a consequence of thermal activation. A peak at 1178 cm^‒1^, attenuated to the Si‒O‒Si asymmetric vibrational frequency, confirms the presence of silica skeleton in Turkish perlite. After thermal activation, this peak gets shifted to 1227 cm^‒1^, which is a common phenomenon in amorphous silica samples [60]. An intense peak around 1630 cm^‒1 ^in Turkish perlite, characteristic of bending mode (δ_O‒H_) of water molecule[61,62]again highly decreased in TAP. 

**Figure 6:**
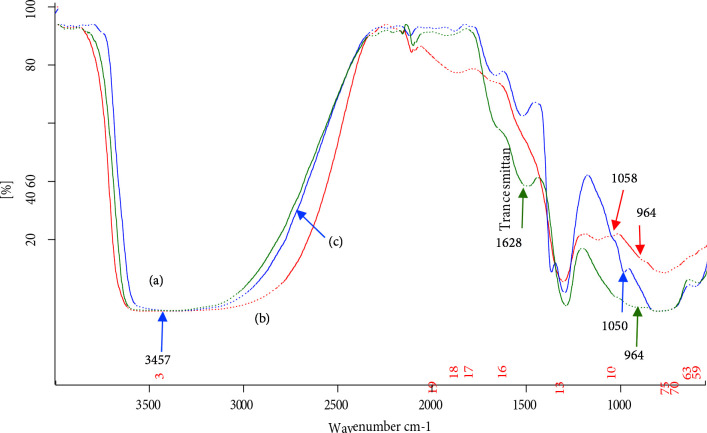
FT‒IR spectra of (a) NPC‒10, (b) NPC‒15 and (c) NPC‒50.

While loading nickel oxide on TAP, its surface gets activated by hydroxyl groups because aqueous NaOH was used as a basification agent during catalyst preparation. Consequently, the FT‒IR spectra of NPC‒10, 15, and 50 (Figure 6) show an increase in broadness and peak intensity of the band attributed to –OH groups. A new band appeared around 1050 cm^‒1^ can be assigned to ≡ Si‒O‒Ni stretching vibration[63], which is normally observed in the range of 1100–1000 cm^‒1^, but, here, it cannot be resolved because of its overlap with the absorbance of Si‒O‒Si stretching, appearing in the range of 1300‒1000 cm^‒1^. An absorption band due to surficial Si‒O‒Ni stretching vibration has appeared at 964 cm^‒1^ [64]. It is also found that the intensity of the band was increased to some extent after increasing nickel oxide incorporation. 

**Figure 7:**
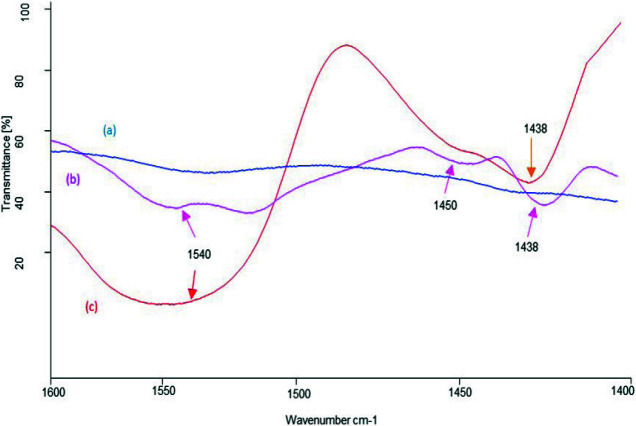
Pyridine adsorbed FT‒IR spectra of (a) NPC‒10, (b) NPC‒15, and (c) NPC‒50.

The FT‒IR spectra of NPC‒10, 15, and 50, obtained after pyridine adsorption in the range of 1550‒1400 cm^‒1 ^are shown in Figure 7. The peak at 1538 cm^‒1^ (Figure 7a) indicates the presence of few Bronsted acidic sites in NPC‒10. In NPC‒15 (Figure 7b), a peak and a broad band appearing at 1438 and 1540 cm^‒1^, respectively[65–67]show the existence of some Lewis and optimal Bronsted acidic sites for suitable catalytic activity. NPC‒50 has the highest Lewis acidic sites corroborated by the appearance of intense peaks at 1438 and 1450 cm^‒1 ^(Figure 7c).

**Figure 8:**
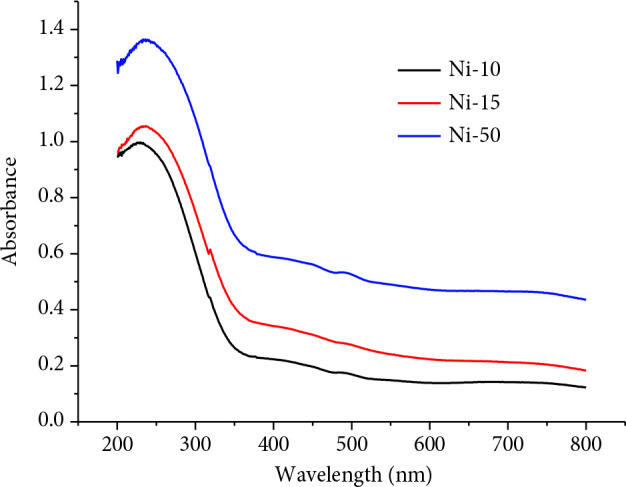
UV‒Vis DRS spectra of NPC‒10, 15, and 50.

UV–Vis DRS is used to determine the state of nickel oxide incorporated into the Si‒O‒Si skeleton of TAP. During steps of synthesis, due to the calcination of catalysts at 450 °C for 4 h, the presence of Ni(OH)_2_ can be declined. A broad band around 250 nm (Figure 8), which becomes more intense with an increase in weight percentage of nickel oxide loading suggests that Ni^+2^ ions are in an octahedral local environment [68].

**Figure 9:**
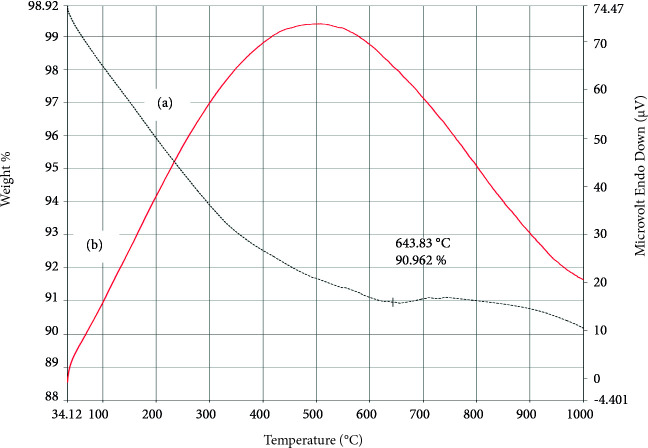
(a) TGA and (b) DSC curve of NPC‒15.

The TGA and DSC curve of NPC‒15 (Figure 9) comprises of two main regions having a major weight loss of about 8.96% up to 643.83 °C and a minor one of approximately 0.82% weight loss at higher temperature along with an exothermic DSC peak. The weight loss in the first region could be due to the removal of bulk water, physisorbed water, and loosely bound hydroxyl groups[69],and decomposition of nickel salt (used as a precursor) [70].The second region shows a minor weight loss, which may be due to the conversion of Ni(OH)_2_ into NiO.

### 3.2. Catalytic activity

The catalytic performance was preliminarily tested by Claisen–Schmidt condensation reaction of cyclehexanone and benzaldehyde to give 2,6-bis(benzylidene)cyclohexanone in a single step, one-pot reaction conditions. The reaction was carried out at 120 °C for 150 min, taking 0.2 g of catalyst, and cyclohexanone/benzaldehyde (molar ratio 1:2). Results given in Table 4 show that Turkish perlite, TAP, and commercial NiO do not possess any catalytic activity for this reaction. In the case of NPC-10, a lower isolated yield (58%) is obtained due to the presence of fewer Bronsted acid sites on the catalytic surface, while, in the presence of NPC‒15, the isolated yield % is highest due to significant increment of active catalytic sites. The isolated yield % is again decreased on using NPC‒50 due to blockage of surface-active Bronsted acidic sites by bulk deposition of NiO crystallites. 

**Table 4:** Catalytic activity of different catalysts for Claisen–Schmidt condensation. reaction.

Catalysts	Isolated yield (%)
Turkish perlite	Nil
TAP	Nil
Commercial NiO	Nil
NPC‒10	58
NPC‒15	90
NPC‒50	48

As depicted from Table 5, NPC‒15 gave a good isolated yield % of 2,6-bis(benzylidene)cyclohexanone in the first as well as in the last run with decent enough consecutive reaction runs when compared with some previously reported catalysts.

**Table 5:** Comparison of results using NPC-15 with some previously reported catalysts for Claisen–Schmidt condensation reaction between cyclohexanone and benzaldehyde.

Catalysts	Reusability of catalyst(No. of runs)	Isolated yield in1st run (%)	Isolated yield in last run (%)
SiO2-R-SO3Ha [71]	10	80	70
40% PW/N-SiO2b [72]	04	98	86
Cs2.5H0.5PW12O40c [73]	05	74	~70
2-HEAAd [74]	-	93	-
NPC-15e[This study]	06	90	83

Based on these results, NPC‒15 was chosen as the main catalyst for catalyzing a series of Claisen‒Schmidt reactions (Table 6). In this series, the reactions of cyclohexanone and various aromatic aldehydes were studied for different periods at 110‒140 °C taking 0.2 g of catalyst and cyclohexanone/aromatic aldehydes (molar ratio 1:2).



**Table 6:** NPC‒15 catalyzed Claisen–Schmidt reaction between cyclohexanone and different aromatic aldehydes.

Product name	Delta value (δ)
5a, 2,6-Dibenzylidenecyclohexanone	7.7 (s, 2H), 7.23-7.55 (m, 10H), 2.82-2.98 (m, 4H), 1.72-1.88 (m, 2H).
5b, 2,6-Di(p-methylbenzylidene)cyclohexanone	7.69 (s, 2H), 7.1-7.31 (m, 8H), 2.82-2.86 (t, 4H), 2.30 (s, 6H), 1.68-1.78 (p, 2H).
5c, 2,6-Di(p-methoxybenzylidene)cyclohexanone	7.66 (s, 2H), 6.88-7.26 (m, 8H), 3.68 (s, 6H), 2.72-2.84 (t, 4H), 1.68-1.8 (p, 2H).
5d, 2,6-Di(p-chlorobenzylidene)cyclohexanone	7.64 (s, 2H), 7.26-7.46 (m, 8H), 2.7-2.82 (t, 4H), 1.67-1.78 (p, 2H).
5e, 2,6-Di(o-chlorobenzylidene)cyclohexanone	7.81 (s, 2H), 7.16-7.28 (m, 8H), 2.67-2.76 (t, 4H), 1.68-1.8 (p, 2H)
5f, 2,6-Di(p-nitrobenzylidene)cyclohexanone	7.9 (s, 2H), 7.4-7.6 (m, 8H), 2.74-2.86 (t, 4H), 1.72-1.86 (p, 2H).
5g, 2,6-Dicinnamylidenecyclohexanone	6.78-7.21(m, 16H), 2.62-2.76 (t, 4H), 1.68-1.78 (p, 2H).

### 3.3. Proposed reaction mechanism

**Scheme 3:**
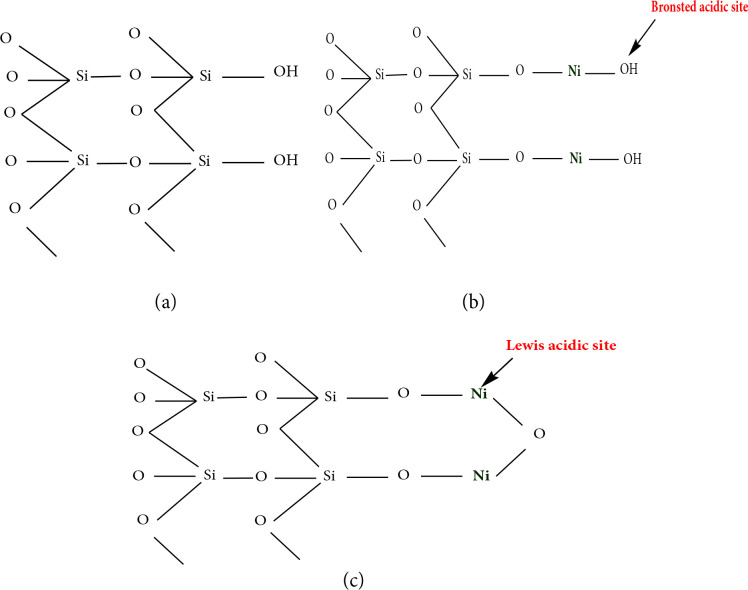
Proposed models of (a) Turkish perlite, (b) NPC‒10, 15 and (c) NPC‒50.

**Scheme 4:**
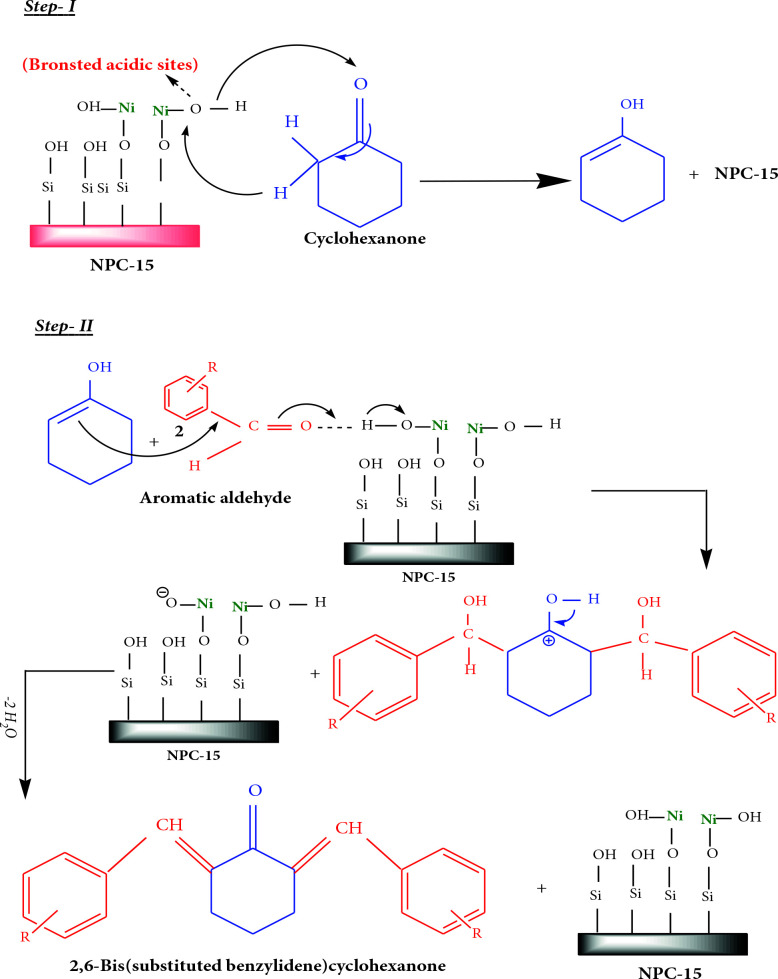
Proposed mechanism of condensation of cyclohexanone and aromatic aldehydes over NPC‒15.

Based on results drawn from XRD, FT-IR, pyridine adsorbed FT-IR, UV-Vis DRS studies, models can be proposed for Turkish perlite, NPC‒10, 15, and 50 catalysts (Scheme 3). They show the presence of Bronsted and Lewis acid sites on the surface of catalysts. The possible pathway for the production of 2,6-bis(substituted benzylidene)cyclohexanones by condensation of cyclohexanone and aromatic aldehydes catalyzed by NPC‒15 is shown in Scheme 4. The surface Bronsted acidic sites of NPC‒15 initiate the reaction by ketone protonation (protonation of carbonyl functional group) followed by abstraction of a proton from α-carbon of the ketone, and active enol intermediate form is produced. Thus, intermediate form condenses with aromatic aldehydes via nucleophilic addition to form desired products and releases water as a side product.

### 3.4. Regeneration of catalyst

**Figure 10:**
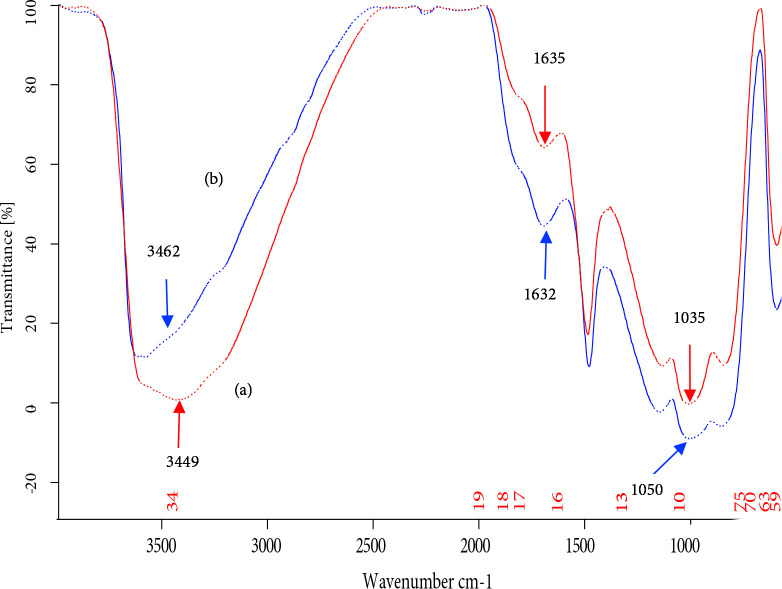
FT‒IR spectra of (a) fresh NPC‒15 and (b) regenerated NPC‒15 after the sixth reaction cycle.

The spent catalyst was regenerated by doing simple filtration, washing with acetone, drying at 110 °C in an oven for 12 h, and finally by calcination at 450 °C in a muffle furnace for 1 h. Thus, obtained regenerated catalyst showed efficient catalytic activity up to consecutive 6 reaction cycles giving almost similar isolated yield % in the range of 90%–83%, indicating the presence of stabilized acidic sites in the regenerated catalyst. After the sixth reaction cycle, the yield % was decreased significantly, this may be due to blockage of acidic sites of the catalyst by the deposition of carbonaceous residues of organic reactants and products on the surface. The FT‒IR spectrum (Figure 10) of regenerated NPC‒15 regenerated after the sixth reaction cycle is similar to that of fresh NPC‒15 demonstrating the stability of nickel oxide loading on Turkish perlite. 

### 3.5. Product identification by 1H-NMR (CDCl3, 500 MHz)

All the products synthesized by Claisen–Schmidt condensation reactions were identified by melting point analysis and ^1^H-NMR spectroscopy. δ-values of all synthesized products are summarized in Table 7. 

**Table 7:** Product Identification by 1H-NMR (CDCl3, 500MHz).

Product name	Delta value (δ)
5a, 2,6-Dibenzylidenecyclohexanone	7.7 (s, 2H), 7.23-7.55 (m, 10H), 2.82-2.98 (m, 4H), 1.72-1.88 (m, 2H).
5b, 2,6-Di(p-methylbenzylidene)cyclohexanone	7.69 (s, 2H), 7.1-7.31 (m, 8H), 2.82-2.86 (t, 4H), 2.30 (s, 6H), 1.68-1.78 (p, 2H).
5c, 2,6-Di(p-methoxybenzylidene)cyclohexanone	7.66 (s, 2H), 6.88-7.26 (m, 8H), 3.68 (s, 6H), 2.72-2.84 (t, 4H), 1.68-1.8 (p, 2H).
5d, 2,6-Di(p-chlorobenzylidene)cyclohexanone	7.64 (s, 2H), 7.26-7.46 (m, 8H), 2.7-2.82 (t, 4H), 1.67-1.78 (p, 2H).
5e, 2,6-Di(o-chlorobenzylidene)cyclohexanone	7.81 (s, 2H), 7.16-7.28 (m, 8H), 2.67-2.76 (t, 4H), 1.68-1.8 (p, 2H)
5f, 2,6-Di(p-nitrobenzylidene)cyclohexanone	7.9 (s, 2H), 7.4-7.6 (m, 8H), 2.74-2.86 (t, 4H), 1.72-1.86 (p, 2H).
5g, 2,6-Dicinnamylidenecyclohexanone	6.78-7.21(m, 16H), 2.62-2.76 (t, 4H), 1.68-1.78 (p, 2H).

## 4. Conclusion

15 wt. % nickel oxide loaded on Turkish perlite (NPC-15) was proven to be an effective and competent solid acid catalyst, possessing significant Bronsted acidity to catalyze condensation reactions between cyclohexanone and aromatic aldehydes with high isolated yield (90%–93%) of desired products in one-pot, liquid phase, solvent-free reaction conditions. The catalyst was easily filtered, regenerated, and recycled several times with analogous efficiency, suggesting the stability of acidic sites of the catalyst during the reactions. The results of X-ray diffraction (XRD), Brunauer–Emmett–Teller (BET) surface area, pyridine adsorbed Fourier-transform infrared (FT-IR), etc. recommended that the optimum catalytic activity of NPC-15 was due to the presence of sufficient surface-active Brönsted acidic sites. While, at higher wt. % loading, NiO crystallites increased Lewis acidic sites and blocked the active Bronsted acidic sites, which ultimately decreased isolated yield % of desired products. XRD results depicted that, in NPC-15, nickel oxide particles were finely dispersed on the Turkish perlite surface in the amorphous phase. A fine dispersion of nickel oxide particles was also shown in scanning electron microscope (SEM) and transmission electron microscope (TEM) images of NPC-15. The novelty of this work is the utilization of abundant natural waste, Turkish perlite as solid support for the synthesis of highly efficient heterogeneous acid catalysts. This investigation also suggests that Turkish perlite could be an alternative to commercial silica for synthesizing novel solid acid catalysts, which can catalyze various industrially important reactions in a cost-effective manner.
